# Control of Dendritic Morphogenesis by Trio in *Drosophila melanogaster*

**DOI:** 10.1371/journal.pone.0033737

**Published:** 2012-03-15

**Authors:** Madhuri Shivalkar, Edward Giniger

**Affiliations:** Axon Guidance and Neural Connectivity Unit, Basic Neuroscience Program, National Institute of Neurological Disorders and Stroke, National Institutes of Health, Bethesda, Maryland, United States of America; VIB and KU Leuven, Belgium

## Abstract

Abl tyrosine kinase and its effectors among the Rho family of GTPases each act to control dendritic morphogenesis in *Drosophila*. It has not been established, however, which of the many GTPase regulators in the cell link these signaling molecules in the dendrite. In axons, the bifunctional guanine exchange factor, Trio, is an essential link between the Abl tyrosine kinase signaling pathway and Rho GTPases, particularly Rac, allowing these systems to act coordinately to control actin organization. In dendritic morphogenesis, however, Abl and Rac have contrary rather than reinforcing effects, raising the question of whether Trio is involved, and if so, whether it acts through Rac, Rho or both. We now find that Trio is expressed in sensory neurons of the *Drosophila* embryo and regulates their dendritic arborization. *trio* mutants display a reduction in dendritic branching and increase in average branch length, whereas over-expression of *trio* has the opposite effect. We further show that it is the Rac GEF domain of Trio, and not its Rho GEF domain that is primarily responsible for the dendritic function of Trio. Thus, Trio shapes the complexity of dendritic arbors and does so in a way that mimics the effects of its target, Rac.

## Introduction

Dendrites are the receptive units of neurons, dictating their connectivity and utility by their specialized, often elaborate shapes. Thus, understanding dendritic morphogenesis - dendritic branching pattern, complexity, field size and targeting - is a key to comprehending the precise networking and efficient functioning of the nervous system. Extensive research in vertebrates and in *Drosophila* has revealed a variety of molecules that influence different features of dendritic morphogenesis [1], [2], [3]. The repertoire of molecules implicated in regulating dendritic growth and development is strikingly varied, including transmembrane receptors, signaling molecules and transcription factors, indicating a multilevel regulation of dendritic morphogenesis. In *Drosophila*, for example, the alternative expression of two transcription factors, Abrupt and Knot, specifies the development, respectively, of simple Class I vs elaborate Class IV multi dendritic - dendrite arborization (md-da) neurons of the peripheral nervous system (PNS), while the interaction of Knot with a third transcription factor, Cut, further discriminates Class III md-da neurons (those with spiky, actin-rich dendrites) [4], [5], [6]. Similarly, transmembrane molecules like Turtle and the cadherin family protein Flamingo (Celsr2) also control dendritic branching and complexity of the same md-da neurons [7], [8]. Thus, dendritic arborization is regulated at different cellular levels.

The small GTPases Rho, Rac and Cdc42 are among the key signaling proteins that regulate dendritic growth and complexity. Rac1, for example, works with a non-canonical Wnt pathway along with β-catenin to promote dendritic branching and growth in rat hippocampal neurons [9], [10]. In *Drosophila*, *rac1* and its paralogs modulate dendritic complexity and field size in mushroom body neurons of the central nervous system (CNS) [11]. In the peripheral nervous system (PNS), *rac1* mutant clones develop a reduced number of dendritic branches in Class IV md-da sensory neurons [12], and ectopic expression of *rac1* promotes branch initiation in all md-da neurons [5], [13]. Despite this evidence for Rac function in dendritic branching its direct regulators and downstream targets in this process are not known. RhoA also is known to regulate dendritic morphogenesis in the fly, for example by limiting dendrite growth in the mushroom bodies of the central brain [14]. Here again, our understanding of its regulation remains incomplete.

In *Drosophila* axons, one key regulator of Rho GTPases, particularly Rac, is the guanine nucleotide exchange factor (GEF), Trio. GEFs are activators of GTPase signaling, catalyzing exchange of GDP for GTP and thereby providing temporal and spatial regulation of GTPase function. While not required for Rac function in epithelial morphogenesis or myotube formation, Trio is essential for Rac activity in axon growth and guidance in the embryo, and in developing adult photoreceptors [15], [16], [17], [18] and *trio* mutant clones display aberrant axon projections in the mushroom body of the adult central brain. Furthermore, *trio^−^* clones in the mushroom body show overextended neurites in the dendritic region of the calyx somewhat similar to those in *RhoA* mutants (though the axonal or dendritic identity of these neurites remains ambiguous) [15].

*Drosophila* Trio, like its *C. elegans* and mammalian orthologs, is a multi-domain protein containing two distinct GEF domains, GEF1 and GEF 2, each characterized by a dbl homology (DH) domain associated with a pleckstrin homology (PH) domain. *trio* genes also share a conserved spectrin repeat, though *Drosophila* Trio lacks a protein serine, threonine kinase domain found in the mammalian protein. Both human and fly Trio selectively interact with Rac GTPases *in vitro* through their GEF1 domains [17], [19], while in human Trio, GEF2 selectively acts on Rho. Activity of the GEF1 domain, but not the GEF2 domain, is essential for growth and guidance of photoreceptor and motor neuron axons in *Drosophila* [17], [18], while GEF2 is required for processes like neurotransmission and pharynx pumping in *C. elegans* [20], [21]. In the fly, moreover, reduction of *trio in vivo* suppresses the rough eye phenotype caused by gain of function of Rac but not of Rho [22], and, consistent with this, GEF activity of the *Drosophila* GEF2 domain has not been demonstrated *in vitro*.

Trio is a particularly attractive candidate for a potential regulator of Rac in dendritic morphogenesis because it is also associated with the Abl tyrosine kinase signaling network, which itself plays a central role in dendritic development in *Drosophila* [23], [24]. Mutant clones of a downstream antagonist of Abl, the actin polymerization factor Enabled (*ena*) exhibit simplified dendritic structures in all md-da neurons in the *Drosophila* PNS; conversely, loss of *Abl* activity increases the number of dendritic branches, while cell specific over-expression of *Abl* in the same neurons reduces dendritic branches [24]. *trio* was originally isolated genetically as an enhancer of the *Abl* mutant phenotype, showing dosage-sensitive genetic interactions with Abl pathway genes in various axon growth and guidance assays and for organismal viability, and this led to its assignment as a core component of the Abl pathway [25].

Given that both Rho GTPases and Abl are potent regulators of dendritic morphogenesis, the potential role of Trio as a linker between them in dendrites becomes a critical question. This is particularly true since in many systems Rac and Abl cooperate closely [26], [27], [28], and in some *Drosophila* axons, the GTPase output of Abl/Trio pathway signaling has been shown to be executed selectively by Rac [17], [18]. In dendritic branching, however Rac and Abl evidently have opposite effects: Rac promotes branch formation while Abl inhibits it. Therefore, we cannot predict *a priori* whether Trio is likely to be involved in dendrogenesis, and if so, whether it will behave like Abl to suppress branching, like Rac to induce it, or neither. Here, we investigate the role of Trio in dendritic morphogenesis of md-da sensory neurons of the *Drosophila* PNS. We find that Trio contributes to shaping the dendritic architecture of both Class I and Class IV md-da neurons, and this function is mediated primarily through its Rac GEF domain and not through its Rho GEF domain. Trio increases the number of dendritic branches but tends to reduce branch length, leaving the overall size of the dendritic field and the total dendritic length largely unchanged. Trio also affects higher order branches selectively, suggesting its role is largely focused on regulation of these more dynamic, actin-rich dendritic branches.

## Materials and Methods

### Fly stocks

All flies and crosses were grown at 25°C. The following fly stocks were used: *Gal4^2–21^*, *Gal4^2–21^UAS-GFP*, *Gal4^4–77^-UAS-GFP*, *Gal4^109(2)80^-UAS-GFP*, *ppk-Gal4*, *ppk-eGFP*, *trio^1^/TM6b T8Z*, *trio^M89^*, *UAS-trio*, *UAS-* trio^GEF1mu^ */TM6B*, *UAS-* trio^GEF2mu^ *]*. Class-specific *GAL4* lines and marker lines were provided by Fen-Biao Gao (U. Mass. Med. Center, Worcester, MA); *Rac1^J11^Rac2^β^ P[FRT2A] mtl^Δ^/TM6B* was provided by Liqun Luo (Stanford Univ, Palo Alto, CA). As *trio* mutations and UAS-*trio* were on different chromosome, we used two different combinations of Class IV neuron markers for analyzing the *trio* mutant and UAS-*trio* phenotype. The line used as a control for UAS-*trio* over-expression, Gal4^4–77^-UAS-GFP/*ppk*-eGFP, had considerably fewer branches than the control line for *trio* mutations, *ppk*-Gal4; Gal4^4–77^-UAS-GFP. All the control lines (including Class I marker line Gal4^2–21^UAS-GFP) were tested in the heterozygous state.

### Immunofluorescence

Third instar larvae were dissected and blocked with 4% paraformaldehyde (PFA) in 0.3% PBT for 10 mins. The fixed larvae were rinsed and washed thrice for 20 mins each in 0.3% PBT. They were then blocked with 10% donkey serum for 2 hrs at room temperature. Blocked larval fillets were stained with primary antibodies overnight at 4°C. They were then washed thrice at room temperature with 0.3% PBT for 20 mins each and stained with secondary antibodies at RT for 2 hrs. The samples were then again rinsed and washed for 20 mins each with 0.3% PBT at RT and mounted in Vectashield. We used the following antibodies: mouse anti-Trio (1∶250), guinea pig anti-Knot (1∶1000) (a kind gift from A. Moore), mouse anti-Abrupt (1∶5), Alexa 568 anti-mouse (1∶1000), Texas red anti guinea pig (1∶1000). If not otherwise specified, primary antibodies were obtained from the Developmental Studies Hybridoma Bank, and secondary antibodies were from Jackson ImmunoResearch or Invitrogen.

### Microscopy

Late third instar larvae were rinsed in 1× PBS and mounted in 70% glycerol by pressing a cover-slip over them and visualized immediately. To minimize variance, only one segment (sixth abdominal segment) was analyzed for all quantification. All the images were acquired as a series of sections of ∼0.550 µm by Zeiss Axiovision microscope at 20× or 40×.

### Image Processing

For Class IV neurons, a series of images were taken of each quarter of the neuron and their maximum projections were processed and stitched together in adobe photoshop. One single stitched image was then opened in the Neurolucida program (Biosciences) for tracing the dendritic branches. The z-series of each image was used as reference while tracing the dendritic arbor. The traced images were then imported in Neurolucida explorer and analyzed for number of branches and dendritic length. The Image J program was used to analyze the total dendritic arbor area of each Class IV neuron by the polygon method [29]. Image J was also used for measuring the dendritic length of Class I neurons. In the case of vpda neurons, only the dorsal primary branch was used for quantification of dendrites to simplify the analysis. All measurements were stored and quantified in Microsoft Excel. T-test was used to compare two sets of data.

## Results

The md-da sensory neurons of the peripheral nervous system (PNS) cover the entire body wall of *Drosophila* larvae and have a stereotypic arrangement in each abdominal segment. These neurons have been grouped in four Classes depending on their dendritic complexity, starting with very simple “Class I” neurons, to very complex “Class IV” neurons [29], [30]. We have used this system to investigate the function of Trio in dendritic morphogenesis in the present study.

### Trio is expressed in the peripheral sensory neurons of Drosophila

**Figure 1:**
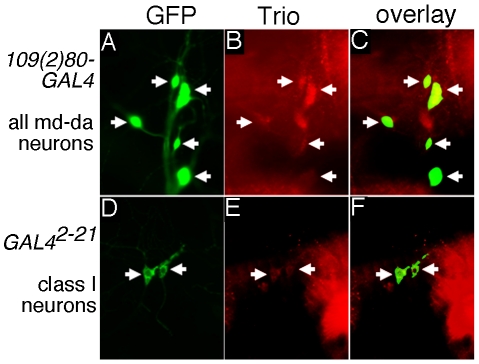
Trio is expressed in PNS sensory neurons. Trio staining (arrows) in dorsal cluster sensory neurons from (**a–c**) 109(2)80 Gal4- UAS-GFP larvae highlighting all md-da neurons and (**d–f**) Gal4 2–21 UAS-GFP larvae highlighting Class I neurons. All the md-da sensory neurons express Trio. While it is readily detected in the cell body and proximal dendritic branches, the signal in small, higher order dendritic branches is difficult to discriminate from immunofluorescence deriving from Trio in associated epithelial cells. Scale bar 20 µm.

Trio is expressed in a variety of tissues and can be detected in developing neuroblasts as early as embryonic stage 10. It is strongly expressed in the CNS, muscle attachment sites and leading edge cells during dorsal closure at later stages of embryonic development [15], [22], [25]. However, expression of Trio in the PNS has not been described [15], [22], [25]. Co-labeling of two md neuron marker lines with anti-Trio antibodies demonstrated that Trio is expressed in all four Classes of md-da neurons (Fig. 1a–c). In the dorsal cluster of sensory neurons, for example, Trio expression is clearly detected in both the Class I neurons, ddaD and ddaE, the Class IV ddaC neuron, as well as in other md-da neurons (Fig. 1d–f). In these cells, Trio is most readily detected in the cell body and proximal parts of the dendritic tree, but it is difficult to assess in the thin, higher order dendritic branches due to immunolabeling of the underlying epithelial cells. Thus, Trio is expressed in the different Classes of da sensory neurons.

### *trio* mutations affect dendritic morphology of Class I neurons

**Figure 2:**
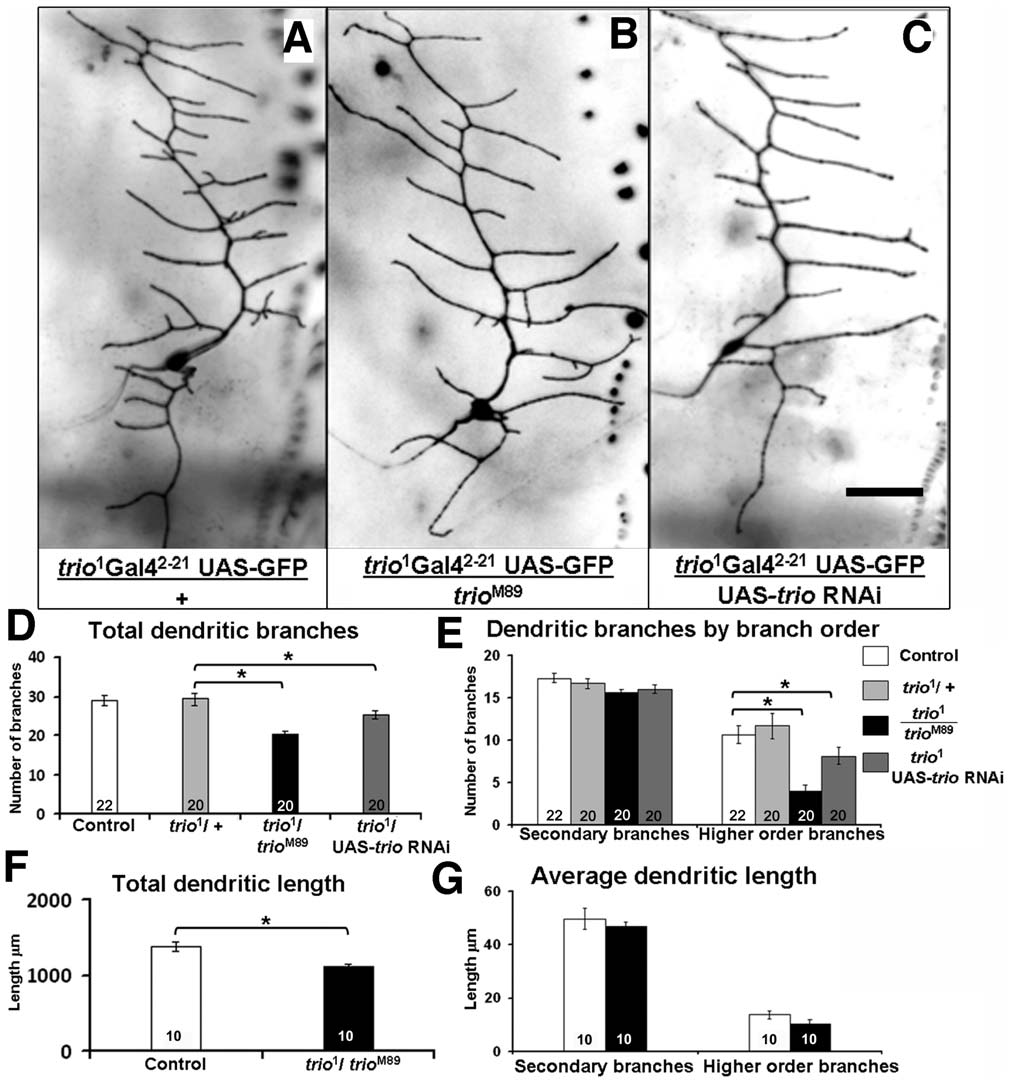
*trio* mutations affect dendritic branching of Class I vpda neurons cell autonomously. **a–c**) A vpda neuron from a wandering third instar larvae of (**a**) *trio* ^1^ Gal4^2–21^UAS-GFP/+ (control), (**b**) *trio* ^1^Gal4^2–21^UAS-GFP/*trio* ^M89^ and (**c**) *trio* ^1^Gal4^2–21^UAS-GFP/UAS-*trio* RNAi; scale bar 50 µm. **d–e**) Quantification of dendritic branching in control, *trio* mutant and UAS-*trio* RNAi expressing vpda neurons. **d**) Dendritic branching of vpda neuron: *trio* knockdown reduces number of dendritic branches, **e**) average number of branches per branch order of vpda neuron: *trio* knockdown affects number of only higher order branches. **f–g**) Quantification of dendritic length in control and *trio* mutant vpda neuron. **f**) Total dendritic length of vpda neuron: *trio* mutations reduce total dendritic length **g**) average dendritic length per branch order of vpda neuron: *trio* mutations do not affect average dendritic length. The number of samples (n value) for each genotype is indicated by the number inside the respective bar, error bars represent standard error of the mean (SEM) and asterisks indicate p value<0.05.

Loss of function experiments reveal that Trio is required for proper arborization of Class I sensory neurons. We analyzed the dendritic structure of the larval vpda neuron in the heteroallelic combination *trio* ^1^ */trio* ^M89^ (Fig. 2 a,b). *trio* ^1^ is a null, lethal allele, *trio* ^M89^ is a hypomorph, and the heteroallelic mutant survives until the pupal stage, simplifying our analysis. Control vpda neurons of the genotype *trio* ^1^ Gal4^2–21^- UAS-GFP*/+* had 29.4±1.6 dendritic branches (mean ± SEM; n = 20) whereas the mutant vpda had considerably fewer, 20.4±0.8 (n = 20, p<0.05) (Fig. 2d). Since the characteristic overall appearance of the vpda neuron was not obviously altered, we checked if the increase in total number of branches was evenly distributed in all orders of dendrites. The number of primary branches and secondary branches (16.7±0.5, n = 20 in control vs. 15.6±0.4, n = 20, p>0.05, in mutants) was not affected. However, the average number of higher order branches decreased considerably, from 11.7±1.5, n = 20 to 4±0.7, n = 20, (p<0.05) in mutant vpda neurons (Fig. 2e). Thus, the decrease in total number of branches was solely attributed to a reduction in higher order branches.

The reduction in branch number was reflected in reduced dendritic length (Fig. 2f). The total dendritic length of the mutant vpda neuron was 1116.8±33.5 µm (n = 10) relative to the control vpda neuron (1371.1±63.1 µm, n = 10, p<0.05). The average length of branches, however, was not altered (Fig. 2g). This was true of primary branches (354.2±18.2 µm in control and 327.2±13.0 µm, n = 10, in mutants), secondary branches (49.7±3.9 µm in control and 47.0±1.4 µm, n = 10, in mutants) and the higher order branches (13.7±1.4 µm in control and 10.3±1.5 µm, n = 10, in mutants). Thus, the reduction in total length was exclusively due to reduction in number of branches.

To determine whether Trio is required cell autonomously for dendritic morphogenesis, we expressed UAS-*trio* RNAi selectively in Class I neurons in *trio* ^1^ heterozygous larvae. Neither *trio^1^* heterozygotes (as noted above) nor wild type larvae expressing *trio* RNAi (31.6±1.6 branches, n = 20) showed any change in dendritic structure of the vpda neuron. In contrast, vpda neurons of *trio* heterozygous larvae expressing UAS-*trio* RNAi had significantly fewer dendritic branches (25.2±1.0, n = 20, p<0.05). In this case again, the effect was totally due to decrease in higher order branches (8.1±0.9, n = 20, p<0.05) (Fig. 2c, e). These data confirm that Trio functions cell-autonomously to shape dendritic structures of Class I sensory neurons.

### *trio* mutations reduce dendritic branching of Class IV neurons without affecting dendritic field size

**Figure 3:**
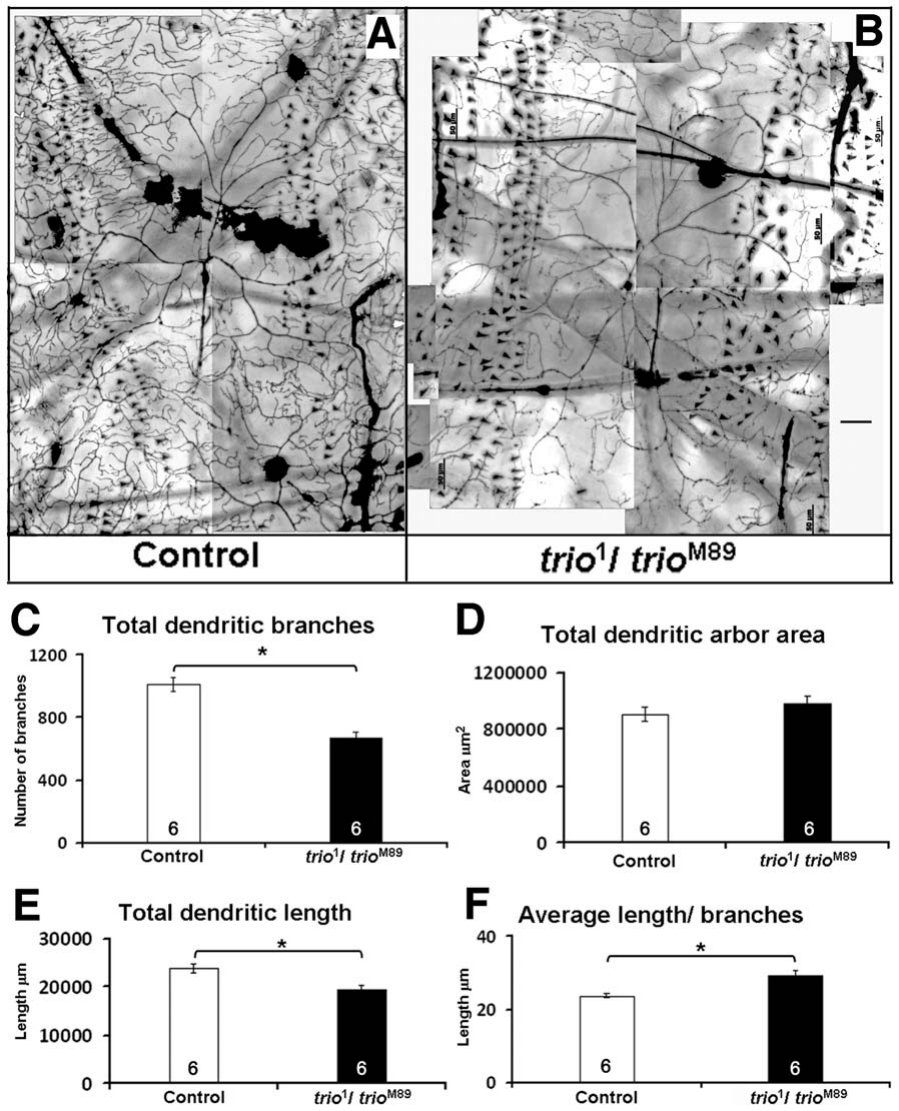
*trio* mutations affect dendritic branching of dorsal cluster Class IV ddaC neuron. **a–b**) A ddaC neuron from a wandering third instar larvae of (**a**) Gal4^4–77^-UAS-GFP/*ppk*-Gal4 control, and (**b**) Gal4^4–77^-UAS-GFP/*ppk*Gal4; *trio* ^1^/*trio* ^M89^; scale bar 50 µm. **c–f**) Quantification of different dendritic parameters in control and *trio* mutant ddaC neurons. **c**) Dendritic branching: *trio* mutations reduce dendritic branching. **d**) Dendritic arbor area: *trio* mutations do not affect dendritic arbor area, **e**) Total dendritic length: *trio* mutations reduce total dendritic length. **f**) Average dendritic length: *trio* mutations increase average dendritic length per branch. The number of samples (n value) for each genotype is indicated by the number inside the respective bar, error bars represent standard error of the mean (SEM) and asterisks indicate p value<0.05.

We extended the analysis of *trio* ^1^ */trio* ^M89^ larvae to complex Class IV md-da neurons and again found a significant reduction in the number of dendritic branches (Fig. 3 a–c). Control ddaC neurons had 1011.5±45.0, n = 6 dendritic branches, whereas the *trio* mutant ddaC neuron had 668±38.6 (n = 6, p<0.05) (Fig. 3c). In this case also, the reduction in total dendritic branches was reflected in total length (Fig. 3e). The total dendritic length of the control ddaC neuron was 23802.1±859.0 µm (n = 6) whereas that of the mutant ddaC neuron was 19474.5±879.4 µm (n = 6, p<0.05). As the total length is a product of number of branches and their length, we wanted to know if the average length of branches was also affected. We therefore measured average branch length, finding that the average length in control ddaC neurons was 23.6±0.6 µm (n = 6), whereas in *trio* mutants it was significantly increased to 29.3±1.2 µm (n = 6, p<0.05) (Fig. 3f). Thus, Trio affected dendritic branching differently from length in Class IV neurons. Despite these profound changes in dendritic number and length, the total area covered by the ddaC dendritic arbor was unchanged in the mutants (984631.2±54214.1 µm^2^ (n = 6) in the mutant compared to 908170±48866.1 µm^2^ in controls (n = 6, p>0.05); Fig. 3d).

### Analysis of *trio* over-expression in Class I neurons

**Figure 4:**
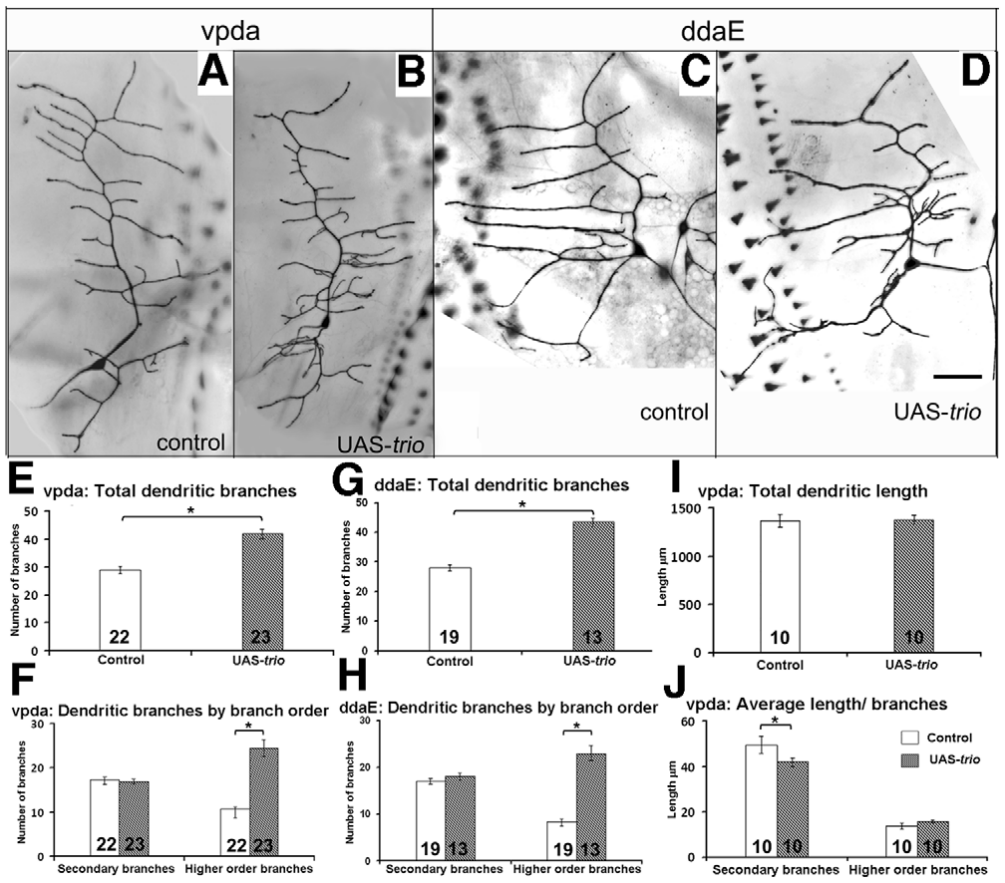
*trio* over-expression in Class I neurons. **a–d**) third instar larval Class I neurons in control Gal4^2–21^ UAS-GFP/+ (**a**) vpda, (**c**) ddaE, and Gal4^2–21^ UAS-GFP/UAS-*trio* (**b**) vpda, (**d**) ddaE; scale bar 50 µm. **e–j**) Quantitative analysis of dendritic parameters in control and UAS-*trio* expressing vpda and ddaE neuron. **e,g**) Dendritic branches: UAS-*trio* promotes dendritic branching of vpda (**e**) and ddaE (**g**) neuron. **f,h**) Average number of branches per branch order: UAS-*trio* affects only higher order branches in both vpda (**f**) and ddaE (**h**) neurons. **i**) Dendritic length: UAS-*trio* doesn't alter total dendritic length. j) Average dendritic length by branch order: UAS-*trio* reduces average length only of secondary branches. The number of samples (n value) for each genotype is indicated by the number inside the respective bar, error bars represent standard error of the mean (SEM) and asterisks indicate p value<0.05.

To complement the loss of function analysis, we next examined the consequence of over-expressing wild type *trio* in Class I neurons with the driver GAL4^2–21^. Though the cells retained the characteristic appearance of Class I neurons, all these neurons exhibited an increase in fine branches proximal to the cell body (Fig. 4 a–d). The vpda neurons from control animals had 28.9±1.2 (n = 22) total dendritic branches, whereas *trio* over-expressing vpda neurons showed a significant increase, 41.9±1.8 (n = 23, p<0.05, Fig. 4e). The increase arose selectively from an increase in higher order branches, which more than doubled upon *trio* over-expression: 24.4±1.8 (n = 23) vs. 10.6±1.0 for control (n = 22, p<0.05) (Fig. 4f). In contrast, the number of primary and secondary branches in the control and mutant were comparable, accounting for the maintenance of an overall Class I - like appearance (Fig. 4f). (Note that as branching parameters are highly sensitive to genetic background, quantitative comparisons should be made only within each experiment, and not between different experiments, both here and below).

*trio* over-expression affected the dendritic morphology of a different Class I neuron, ddaE, in the same way as vpda, increasing the total number of dendritic branches (43.4±1.4, n = 13 upon *trio* over-expression vs. 28.1±0.9, n = 19, p<0.05, in controls) without affecting the number of primary branches and secondary branches (Fig. 4g, h). Again, the number of higher order branches increased considerably from 8.3±0.7 (n = 19) to 23±1.6 (n = 13, p<0.05, Fig. 4h) upon *trio* over-expression, and a third Class I neuron, ddaD, showed similar phenotypes as did vpda and ddaE (data not shown). The primary and secondary branches of Class I neurons are stable, microtubule- rich branches whereas the higher order branches are more actin- rich and dynamic [13], [24], [31], and it is these higher-order branches that are sensitive to gain and loss of *trio*.

### *trio* over-expression limits dendritic length

We wondered whether the 50% increase in number of branches upon *trio* over-expression would be reflected in increased dendritic length of the Class I neurons. We, therefore, measured the total length of the dendrites of the vpda neuron in *trio* over-expressing larvae and found that it was not significantly different than that of the control (1381±43.1 µm for *trio* over-expression (n = 10), vs. 1371±63.1 µm (n = 10, p>0.05) for control (Fig. 4i). The increase in number of branches, without a change in total length, implied that the average length of branches was compromised. Further analysis of the average dendritic length by branch orders showed no difference in the average length of the primary branches (346.9±8.9 µm, n = 10) compared to the control (354.2±18.2 µm, n = 10, p>0.05) or of the highest order branches (15.68±0.8 µm, n = 10) compared to the control (13.74±1.4 µm, n = 10, p>0.05) upon *trio* over-expression (Fig. 4j). However, the average length of the secondary branches was significantly reduced to 42±1.9 µm (n = 10) from 49.7±3.9 µm (n = 10, p = 0.0491) in control (Fig. 4j). Thus, *trio* over-expression inhibits the average dendritic length of the second order branches in a way that compensates for the increased number of branches and restores the total length of the dendritic arbor. Altogether, *trio* over-expression affects dendritic branching and growth in an opposite manner by promoting dendritic branches and inhibiting dendritic length and thus preserving the total dendritic length as well as the overall dendritic structure of the Class I neurons.

### *trio* over-expression in Class IV neurons mimics its effect in Class I neurons

**Figure 5:**
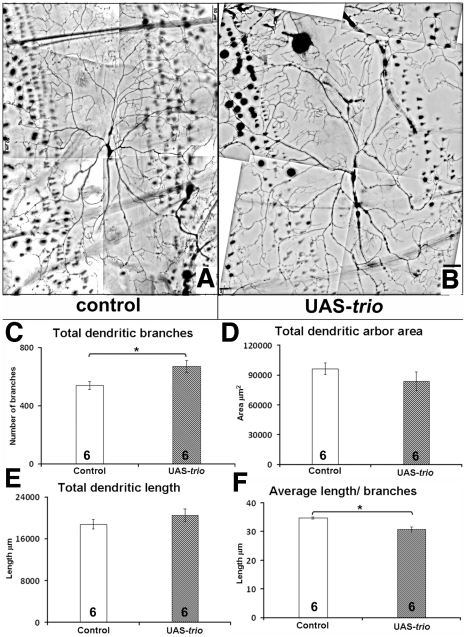
*trio* over-expression in Class IV neuron. **a,b**) third instar larval Class IV ddaC neurons in control Gal4^4–77^UAS-GFP/*ppk*-eGFP and UAS-*trio*/+; Gal4^4–77^UAS-GFP/*ppk*-eGFP; scale bar 50 µm. **c–f**) Quantitative analysis of dendritic parameters in control and UAS-*trio* expressing ddaC neuron. **c**) Dendritic branches: *trio* over-expression promotes dendritic branching of ddaC neuron. **d**) Dendritic arbor area: *trio* over-expression does not affect dendritic arbor area **e**) Dendritic length: *trio* over-expression doesn't alter total dendritic length. **d**) Average dendritic length: *trio* over-expression reduces average dendritic length per branch. The number of samples (n value) for each genotype is indicated by the number inside the respective bar, error bars represent standard error of the mean (SEM) and asterisks indicate p value<0.05.

Consistent with the observations above, over-expression of *trio* in Class IV neurons with class specific GAL4 driver and GFP reporter (*Gal4^4–77^ and ppk-eGFP*) also increased the number of dendritic branches (Fig. 5 a–c). For example, for the dorsal Class IV neuron, ddaC, the number of branches was 669.3±41.9 branches with *trio* over-expression (n = 6), vs. 540.3±26.5 for control (n = 6, p<0.05) (Fig. 5 c). In contrast to the loss of function, this was not accompanied by any change in total dendritic length (20554.6±1228.0 µm, n = 6) vs. control (18792.1±917.0 µm, n = 6, p>0.05) (Fig. 5 e). These data implied that, as in Class I neurons, the average length of dendrites may be compromised upon *trio* over-expression. Accordingly, the average length of dendrites was reduced to 30.7±0.8 µm (n = 6, p<0.05) upon *trio* over-expression from 34.7±0.2 µm (n = 6) in the control (Fig. 5 f). This reduction in average length was not restricted to any particular set of branch orders but affected all the dendritic branches from primary to higher order branches (data not shown). Similar to the loss of function, the average arbor area of ddaC was not affected by *trio* over-expression, (83695.2±9268.2 µm^2^, for *trio* over-expression n = 6 vs. (96564.91±5920.8 µm^2^, for control n = 6), p>0.05) (Fig. 5 d). Altogether, *trio* over-expression affects both Class I and Class IV neuron morphology in similar ways, consistently promoting branching while often inhibiting various aspects of dendritic length, and maintaining overall arbor area.

### Trio function in Class I neurons is mediated via Rac GTPases

**Figure 6:**
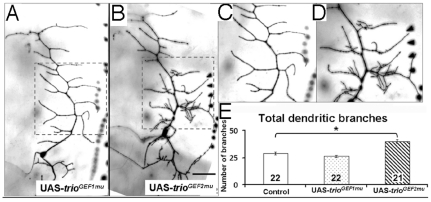
Trio acts via its GEF1 domain to regulate dendrogenesis. **a,b**) Third instar larval vpda neuron expressing (a) UAS- *trio^GEF1mu^* and (b) UAS- *trio^GEF2mu^*; scale bar 50 µm **c–d**) magnified view of dendritic branching (**c**) and (**d**) are highlighted areas in (a) and (b). **e**) Quantitative analysis of dendritic branching: ectopic expression of UAS- *trio^GEF2mu^* but not UAS- *trio^GEF1mu^* promotes formation of extra dendritic branches. The number of samples (n value) for each genotype is indicated by the number inside the respective bar, error bars represent standard error of the mean (SEM) and asterisks indicate p value<0.05.

**Figure 7:**
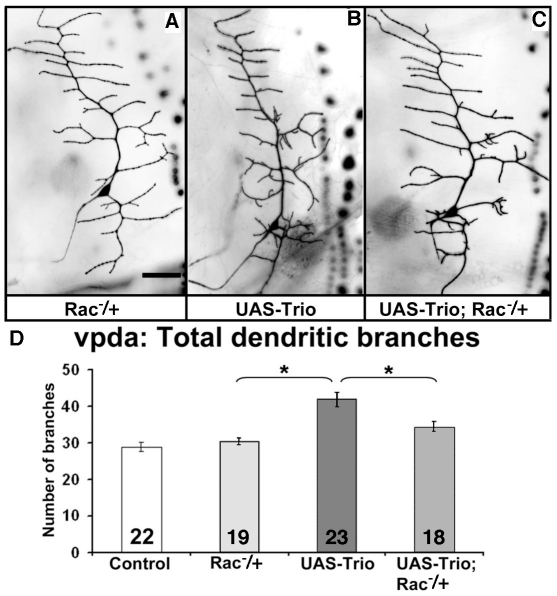
Reducing Rac suppresses the effect of Trio overexpression. Larvae of the indicated genotypes were dissected and vpda branch number was quantified. (A–C) Representative examples of vpda neurons labeled with mCD8-GFP and visualized by fluorescent microscopy. (A) *rac1^J11^rac2^Δ^mtl^Δ^/GAL4^2–21^UAS-mCD8-GFP*, (B) *UAS-Trio; GAL4^2–21^UAS-mCD8-GFP*, (C) *UAS-Trio; rac1^J11^rac2^Δ^mtl^Δ^/GAL4^2–21^UAS-mCD8-GFP*. (D) quantification of vpda branch number. Vertical bars report number of branches for the indicated genotypes, lines indicate SEM. Number in each bar is the number of samples. Asterisks indicate p<0.05.

In the *Drosophila* CNS and PNS, Trio exerts its effects on some axon guidance events selectively via its Rac GEF domain and not via its Rho GEF domain. We tested if Trio has such a preferential downstream signaling in dendritic morphogenesis as well. We over-expressed two different constructs of full length *trio*, each of which had a mutation inactivating either the Rac GEF domain (UAS- *trio^GEF1mu^*) or the Rho GEF domain (UAS- *trio^GEF2mu^*) [17] (Fig. 6 a–d). Over-expression of *trio^GEF1mu^* was not able to alter the total number of dendritic branches of the Class I vpda neuron (26.1±1.1, n = 22 vs 28.9±1.2, n = 22 for control, p>0.05) (Fig. 6 a, e). In contrast, over-expression of *trio^GEF2mu^* construct increased the total dendritic branches to the same degree as did over-expression of wild type *trio* (39.7±1.6, n = 21, p<0.05) (Fig. 3 b, e). As for wild type, the major effect was on the higher order branches (data not shown). These results indicate that Trio contributes to dendritic development primarily through its Rac-specific GEF1 domain, and not with its GEF2 domain. Consistent with this hypothesis, reducing the dosage of the three *Rac* paralogs by 50% significantly suppressed the effect of Trio overexpression on vpda branch number (Figure 7: 34.5±1.3 branches (n = 18) for *GAL4^2–21^; UAS-Trio; [Rac1^J11^Rac2^Δ^Mtl^Δ^]/+ vs* 41.9±1.8 (n = 23) for *GAL4^2–21^; UAS-Trio* (mean±SEM; p<.01); note that [*Rac1^J11^Rac2^Δ^Mtl^Δ^]/+* was not significantly different from control: 30.4±0.9 branches (n = 19)).

### *trio* does not change expression of neuronal Class-specific transcription factors

**Figure 8:**
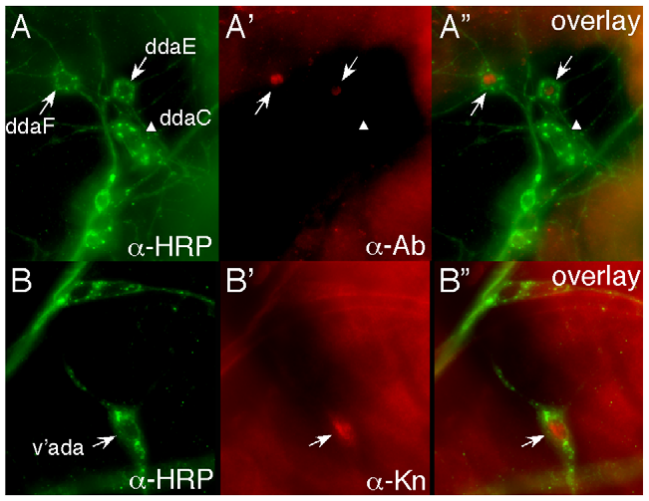
Trio does not transform class identity of md-da neurons. *trio^1^/trio^M89^* mutant larvae were filleted and stained with the pan-neuronal antibody anti-HRP (green) and the indicated antibodies against class-specific markers (red). (A-A″) anti-Abrupt (Class I). Absence of Trio does not prevent expression of Abrupt in the Class I neurons ddaE or ddaF (arrows), nor does it induce expression of Abrupt in the Class IV neuron ddaC (arrowhead). (B-B″) anti-Knot (Class IV). Absence of Trio does not prevent expression of Knot in the Class IV neuron v' ada (arrow).

The over-expression of *trio* changed the complexity of dendritic branching and it was clearly associated with the GEF1 domain of Trio that activates Rac1. Analyses of Rac function in morphogenesis typically focus on its regulation of the cytoskeleton, but Rac can also control transcription through its activation of Jnk and c-Jun. We therefore wondered whether the effects of Trio and Rac on dendritic branching might be due to changes in expression pattern of neuron specific transcription factors such as Abrupt and Knot. These transcription factors have complementary expression patterns in Class I and Class IV neurons, respectively, and they shape the dendritic pattern of the class of neurons they are expressed in [4], [5], [6]. We stained *trio* mutant larvae (*trio^1^/trio^M89^*) with anti-Abrupt and anti-Knot antibodies and found that the pattern of Abrupt and Knot expression was unchanged, with Abrupt expressed only in Class I neurons and Knot only in Class IV neurons (Fig. 8). We also did not detect any change in Abrupt or Knot expression in larvae that overexpressed *trio* derivatives (*trio^GEF1mu^* or *trio^GEF2mu^*) in Class I neurons under control of *GAL4^2–21^* (data not shown). Thus, the effect of *trio* on dendritic branching is not due to altered expression of the transcription factors Abrupt and Knot, with consequent changes in Class I vs. Class IV identity.

## Discussion

Trio has been associated with both Rho family GTPases and the Abl tyrosine kinase. Both these pathways control dendritic arborization in *Drosophila*, but they do so in different ways, with Rac, for example, promoting dendritic branching and Abl limiting it. This made it important to determine whether Trio plays a role in dendrogenesis, and if so, whether it was functioning in association with Rac or with Rho, and how its effects compared with those of Abl. We show here that Trio also shapes dendritic structure in the fly. In both simple Class I sensory neurons and complex Class IV sensory neurons, Trio promotes formation of dendritic branches: over-expression of *trio* produces more elaborately branched dendritic trees whereas loss of *trio* reduces the number of dendritic branches. In both cases, the effect of Trio is concentrated on higher-order branches, which others have shown to be actin-dominated and more dynamic, and not in the primary branches, which tend to be microtubule-dominated and more stable [13], [24], [31].

Trio not only affects dendritic branching but also dendritic length. In most assays, Trio limits the average length of some or all orders of dendritic branches to a degree that roughly offsets the increase in branch number, leading to a modest net change or no change in total dendritic length. The compensation is not exact, however. For example, in *trio* mutants, while average dendritic length is unchanged in Class I neurons, an increase in average branch length is seen in Class IV neurons but it is not enough to counteract the decrease in branch number, leading to an overall decrease in total length. Conversely, in *trio* over-expression, both Class I neurons and Class IV neurons show no net change in total length in spite of an increase in the average length of dendrites. This variability may suggest that total dendritic length is not strictly invariant for a given sensory neuron, with a fixed length parceled among a variable number of branches, but rather that Trio may have separate, and opposite, effects on branch length and number. Further experiments will be necessary, however, to test this idea.

Expression of constructs bearing mutations in each of its GEF domains suggests that Trio acts primarily through its Rac GEF domain, and not its Rho GEF domain, to affect dendritic morphogenesis of the PNS sensory neurons. Thus, a Trio derivative lacking Rac GEF activity does not alter dendritic structure whereas a derivative lacking Rho GEF activity produces effects that are indistinguishable from those of the wild type protein. This is consistent with the similarity between the phenotype we observe for gain and loss of *trio* function and that reported for gain and loss of Rac [5], [12], [13], [32], and also with data from axonal development, both in embryonic motor neurons [18] and adult photoreceptors [17] showing that the Rac-specific GEF1 domain is the key effector domain of Trio in axons. It is in contrast, however, to results from the adult *Drosophila* mushroom body, in which *trio* mutant clones showed overextension of neurites similar to that in *RhoA* mutant clones in the dendritic portion of the structure (the calyx) [15]. Perhaps Trio pairs with different GTPases in different developmental settings, as has been observed for *C. elegans* Trio [20], [21], [33]. Our results also indicate that the dendritic phenotypes seen upon over-expression of *trio* are not due to changes in expression of the important neuronal class specific transcription factors, Abrupt and Knot, thus arguing against the idea that changes of cell fate are responsible for changes in dendritic morphology in these experiments.

In contrast to the concordance between the effects of Trio and Rac, the phenotypes produced by altering Trio activity are opposite to those from manipulation of the Abl tyrosine kinase pathway. This was surprising in light of prior work showing that the effects of Trio mimic those of Abl in axonal development, and that led to the suggestion that Trio is a core component of the Abl pathway [22], [25]. Two hypotheses could account for this discrepancy. First, it could be that the Trio-Rac module should be thought of as an adjunct to the Abl signaling network, with a variable and context-dependent relationship to Abl, rather than as itself being a core element of that pathway. Such a relationship would allow the Trio-Abl interaction to produce different morphological outcomes in different developmental settings. Alternatively, we cannot rule out the possibility that the relationship of Trio to Abl at the molecular level is the same in dendrites as in axons, but it manifests in opposite morphological consequences due to the complexities of the relationship between signaling, cytoskeletal dynamics and morphology. Indeed, there are many examples of a cytoplasmic signaling protein producing seemingly opposite effects in different developmental contexts [18], [34], [35], [36], [37], [38]. In the current setting, however, we do not favor this interpretation since such non-linear effects of signaling proteins in other systems typically lead to observation of contradictory phenotypes upon manipulating the activity of a gene across a wide dynamic range [38]. In the case of Trio, in contrast, all of our gain- and loss-of function manipulations give a consistent set of effects on dendritic branching. Additional experiments will be required, however, to distinguish fully between these hypotheses.

The data reported here show that Trio, like its effector Rac, regulates dendritic arborization in *Drosophila* sensory neurons. Our data also suggest that the relationship of Trio to the Abl tyrosine kinase signaling network may be more nuanced than was previously appreciated. It seems likely that the interplay of these signaling modules channels the molecular machinery of morphogenesis in a variety of ways to help produce the vast range of neuronal shapes.

While we were preparing these results for publication, it came to our attention that Dr. Daniel Cox and co-workers (George Mason University) had independently performed a complementary set of experiments investigating the effects of Trio on dendritic arborization, and had reached very similar conclusions about the effects of Trio on *Drosophila* md-da neurons and its preferential reliance on Rac signaling.
